# Role of SOX2 in the Etiology of Embryonal Carcinoma, Based on Analysis of the NCCIT and NT2 Cell Lines

**DOI:** 10.1371/journal.pone.0083585

**Published:** 2014-01-03

**Authors:** Ronak Eini, Hans Stoop, Ad J. M. Gillis, Katharina Biermann, Lambert C. J. Dorssers, Leendert H. J. Looijenga

**Affiliations:** Erasmus MC, University Medical Center Rotterdam, Department of Pathology, Rotterdam, The Netherlands; University of Tampere, Finland

## Abstract

The transcription factor SOX2, associated with amongst others OCT3/4, is essential for maintenance of pluripotency and self-renewal of embryonic stem cells. SOX2 is highly expressed in embryonal carcinoma (EC), the stem cell component of malignant nonseminomatous germ cell tumors, referred to as germ cell cancer (GCC). In fact, OCT3/4 together with SOX2 is an informative diagnostic tool for EC in a clinical setting. Several studies support the hypothesis that SOX2 is a relevant oncogenic factor in various cancers and recently, SOX2 has been suggested as a putative therapeutic target for early stage EC. We demonstrate the presence of genomic amplification of SOX2 in an EC cell line, NCCIT, using array comparative genome hybridization and fluorescence *in situ* hybridization. Down-regulation of SOX2 by targeted siRNA provokes NCCIT cells towards apoptosis, while inhibition of OCT3/4 expression induced differentiation, with retained SOX2 levels. Mice pluripotent xenografts from NCCIT (N-NCCIT and N2-NCCIT) show a consistent SOX2 expression, in spite of loss of the expression of OCT3/4, and differentiation, with retained presence of genomic amplification. No SOX2 amplification has been identified in primary pure and mixed EC *in vivo* patient samples so far. The data presented in this study are based on a single EC cell line with a SOX2 amplification, with NT2 as control EC cell line, showing no profound induction of apoptosis upon SOX2 downregulation. The findings are of relevance to identify mechanisms involved in the pathogenesis of EC tumors, and support the model of SOX2-oncogene dependency of EC, which however, does not exclude induction of differentiation. This finding is likely related to the presence of wild type p53 in GCC, resulting in expression of downstream target genes, amongst others miR-34a, miR-145 and SOX2, associated to the unique sensitivity of GCC to DNA damaging agents.

## Introduction

SOX2 (sex-determining region Y-box2) is a 317 amino-acid transcription factor containing an HMG domain located at 3q26, being a critical transcription factor of normal embryonic stem (ES) cell development and maintenance, as well as neural stem cells [1], [2]. During early embryogenesis, Sox2 is required for epiblast maintenance, and formation of multipotent cell lineages in early mouse development depends on Sox2 function [3]. Moreover, Sox2 is one of the four transcription factors successfully used to induce pluripotent stem cell (iPS) from mouse and human fibroblast cells [4], [5]. In particular, in these cells SOX2 physically interacts with OCT3/4 and NANOG forming an interconnection machinery that binds to promoters of numerous but defined stem cell genes to induce their expression as well as repress expression of genes related to differentiation [1].

This seems essential since generating iPS cells from primary human fibroblast has become possible with the single use of OCT3/4 and SOX2 [5]. Relative hyper- or hypo-expression of these pluripotency factors may result in aberrant self-renewal of ES cells and can possibly even promote oncogenesis [6]. Recent studies have shown that SOX2 over-expression leads to aberrant stem cell self-renewal signaling in breast cancer cells [7], [8]. Moreover, several studies have shown over-expression of SOX2 in various cancers including glioblastoma [9], non-small cell lung cancer [10], [11], prostate cancer [12] and hepatocellular carcinomas [13] supporting SOX2 as a relevant oncogene in these malignancies. Specifically, SOX2 is reported as a lineage-survival oncogene in squamous cell carcinoma of the lung [14]–[16] and its over-expression is associated with tumor progression and poor clinical outcome in breast cancer [7], [17]. These reports suggest that SOX2 could activate important gene cascades involved in initiation and progression of tumors and maintenance of a poorly differentiated state [18].

Besides in these epithelial cancers, SOX2 has also been proven to be of diagnostic value in the context of human germ cell cancers (GCC) [19]. Testicular GCC originate from either a primordial germ cell (PGC) or gonocyte during early development [20]–[22]. Histologically and clinically, GCC are classified into seminoma (SE) and non-seminoma (NS). They both originate from the same precursor known as carcinoma *in situ* (CIS), also referred to as intratubular germ cell neoplasia unclassified (IGCNU) [20]–[22], [23]. NS can contain both embryonal and extra-embryonal lineages, including embryonal carcinoma (EC), somatic differentiation (teratoma) and extra-embryonal differentiation (choriocarcinoma (CH) and yolk sac tumor (YS)). EC is the malignant ES cell counterpart, in principle able to differentiate into virtually all tissue lineages [24]–[26]. EC cells show a gene expression profile similar to that of ES cells, including high expression of the core pluripotency transcription factors OCT3/4 and SOX2. These transcription factors act in concert to control stem cell self-renewal and pluripotency [27], [28]. OCT3/4 is expressed in CIS, SE and EC. In contrast, SOX2 is expressed in EC but not the precursor lesions and SE and normal germ cells. In addition, SOX2 can be heterogeneously expressed in differentiated nonseminomatous components. Currently, the expression of OCT3/4 and SOX2 are used for the diagnosis of EC while combination of the presence of OCT3/4 and SOX17 is used for the diagnosis of SE [19]. Additionally, a single study [29] using an EC cell line NEC8 model reported SOX2-siRNA induced apoptotic cell death *in vitro* and growth suppression *in vivo*. In view of the similarity between EC and human ES cells, disruption of the orchestrated activity of these transcription factors could possibly induce lethal effects in EC cells [29].

There are various cell lines representing EC tumors i.e. NTera2 (NT2) [30], NCCIT [31], and 2102Ep [32] in which OCT3/4 and SOX2 are highly expressed [33]. As a result of array comparative genomic hybridization (CGH) on multiple EC cell lines, NCCIT cell line, originating from a mediastinal GCC, showed amplification at the long arm of chromosome 3, band q23 including the SOX2 gene locus [34]. In addition, it is shown that NCCIT has no functional p53, while NT2 and 2102ep cell lines contain wild type p53, expressed in a relative low and normal level, respectively [35]. p53 is a transcription factor that plays a key role in cellular defense mechanisms against neoplastic transformation, found to be mutated in a large number of human cancers, for which GCC is an exception [36].

Moreover, it is shown that p53 plays an active role in promoting differentiation of human ES cells and opposing self-renewal by regulation of specific target genes and microRNAs (miRs). miRs are small, non-coding RNAs of 21–23 nucleotides in length that regulate gene expression, generally at a post-transcriptional level [37]. Specific miRs regulate self-renewal and pluripotency in human cells [38].

In this study we explored whether oncogene dependency of the pluripotency gene SOX2 in EC exist, which might explain the biological and clinical difference(s) between different histological subtypes of GCC. Previously we have shown that reduction of OCT3/4 and SOX2 in NT2 cause induction of differentiation [33]. Here, we investigate the effect of reduction of OCT3/4 and SOX2 by means of targeted siRNA in NCCIT cells. In addition, in order to decipher whether specific amplification of pluripotency genes associates with the undifferentiated state of EC, we investigate whether primary GCC including pure EC and mixed NS including EC components, have amplification of OCT3/4 and SOX2 using Fluorescence *In Situ* hybridization (FISH).

## Materials and Methods

### Cell culture and manipulation

Both NT2 and NCCIT cell lines were received as gifts [31], [39], [40]. NT2 and NCCIT cells were maintained in Dulbecco's modified Eagle's medium (DMEM) (Life technologies Europe BV, Bleiswijk, Netherlands) containing 10% fetal bovine serum supplemented with penicillin and streptomycin (10,000 U/ml) at 37°C under 5% CO2. NT2 cells were passaged at 90% confluence while NCCIT were passaged at 80% confluence. Briefly, cells were seeded at the density of in 75 ml flasks. Seeding ratio for NT2 cells and NCCIT cells were 1∶3 and 1∶5 respectively. Incubated cells were harvested with trypsin-EDTA, washed with PBS, centrifuged and resuspended in DMEM medium. Approximately 8000 cells were used to make cytospins. 100 µl of counted cells (diluted in medium) were put on glass slides. In order to put cells on glass slides, Cytospin chambers were used. The slides were set into cytospin machine for 10 min and 500 rpm. Then chambers were removed and glass slides were air dried for at least 2 hours and were stored at −20°C.

### siRNA transfection for SOX2 and OCT3/4

NCCIT cells were transfected with siRNA based SOX2 (s13294; Ambion/Invitrogen, Breda, the Netherlands), siRNA against OCT3/4 (Qiagen,Manchester, UK) [41] and Silencer Negative Control siRNA (4611, Life technologies Europe BV, Bleiswijk, Netherlands) using Lipofectamine 2000 (Life technologies Europe BV, Bleiswijk, Netherlands) in 24 well-plates (Greiner bio-one/Germany) according to the manufacturers' protocol. The transfection ratio (siRNA:Lipofectamine) was 1∶2.

### Protein Isolation and Western blotting analysis

Isolation of protein and Western blotting analysis were essentially performed as previously described [42]. The antibodies are described in Immunohistochemistry (see below). The dilution of antibodies used for western blot was 1∶1000 for both OCT3/4 and SOX2 antibodies. In addition, mouse monoclonal beta-actin (clone AC-15; Sigma Aldrich, St Louis, MO, USA) was used. The blocking solution used was milk (1%). Binding of the primary antibodies was visualized by using IRDYe donkey anti-mouse or donkey anti-goat secondary antibodies and the blots were scanned on the Odyssey infrared imaging system (from LI COR Biosciences, NE, USA).

### Immunohistochemistry (IHC)

Staining was performed on cell lines as well as FFPE tissues. Unfixed cells were incubated for 1 hour at room temperature with the primary antibodies. Formalin-fixed paraffin-embedded (FFPE) tissues (4 µm thick sections) were pretreated by antigen retrieval (Tris (0.001m/EGTA (0.01 m) PH 9.0) [43] after deparaffinization and blocking of the endogenous peroxidase with H_2_0_2_ (3%) (Merck-KG9A; 108597, Darmstadt, Germany). Endogeneous biotine is blocked by Avidin-Biotin blocking kit (Vector, SP-2001, Burlingame, CA 94010, USA). Incubated overnight at 4°C. Antibodies used were SOX2 (1∶250, AF2018; R&D System, USA), OCT3/4 (sc-5279; 1∶350; sc-Santa Cruz Biotechnology, Santa Cruz, CA, USA), Ki67 (1∶50, A047; Dako, CA, USA) and Caspase 3 (1∶500, 9579; Cell Signaling).

Visualization was performed by using horseradish peroxidase avidin-biotin complex (Vectastain, Vector SP-2001, Burlingame, CA 94010, USA) and DAB/H_2_0_2_ as substrate. For negative controls, primary antibody was omitted, resulting in complete absence of signals.

### Percentage of knock-down

Cells were harvested by trypsinization and analyzed for knock-down of the proteins under investigation using IHC on cytospins (see below). Percentage of positive cells for each gene was calculated by counting five different regions on the cytospin in which each region contained 100 cells, therefore, the total number of counted cells were 500 cells. The cells were defined as positive when the color brown was above the background color and cells with color blue above the background level were counted as negative for antibody used. This method has been tested and published in [33].

### Cell viability test

Cell death induced by siRNA transfection was evaluated by trypan blue exclusion. Briefly, NCCIT cells were harvested after 48 h, 54 h and 60 h after siRNA transfections. Cells were washed with PBS and resuspended in 100 µl PBS. After mixing with 100 µl 0.8% trypan blue cells were calculated as the number of blue cells/total number of cells.

### Flow cytometry

#### Propidium Iodide

In order to measure the percentage of live and dead cells, Propidium Iodide (PI) assay was used. Briefly, cell pellets were resuspended in 500 µl of PBS. Cells were fixed by adding 5 ml of 70% cold ethanol. Then the cell suspension was centrifuged at 400 g for 5 min and the supernatant was removed. Cell pellet was washed with PBS and resuspended in 1 ml of PI staining solution (50 µg/ml) according to the standard protocol. Cells were incubated for at least 30 min at room temperature in the dark to be analyzed by flow cytometry using FACSAria III machine using 488-nm laser line for excitation.

#### AnnexinV

Percentage of apoptotic cells was measured using BD Pharmingen FITC AnnexinV apoptosis detection kit I (556547; Erembodegem, Belgium) according to the manufacturers' protocol. Hoechst 33258 (H3569), (Molecular probe/Invitrogen, Leiden, the Netherlands) was used at the concentration of 1 µg/ml. Briefly, harvested cells (1×10 ∧5) were washed with PBS, centrifuged and resuspended in Annexin buffer provided with the kit. The final concentration used for Annexin was 0.5 µg/µl. Cells were analyzed by FACSAria III machine.

### *In Situ* hybridization

Fluorescent *in situ* hybridization (FISH) was performed with probe mapped to chromosome 3 band 26.33 (RP11-43F17) for SOX2 detection. In addition, an OCT3/4 specific probe was used (RP11-1058J10). In addition to this specific region, a probe mapped to centromeric region of chromosome 12 was assessed as control [44]. Briefly, FFPE tissue section of 4 µm thickness were deparaffinized, pretreated in a 0.01 M Sodium citrate solution under high pressure in pressure cooker, subsequently with pepsin (4.000 U) at 37°C followed by washing and dehydration. Probes were labeled by nick-translation, according to standard procedures, either with dioxigenin-11-dUTP (Roche, Manheim, Germany) or biotin-16-dUTP (Roche, Manheim, Germany), and applied in 10-15 µl hybridization mixture on the tissue slides. The probes were denatured together with the target by placing the slide for 10 min on the 80°C oven. After hybridization overnight at 37°C, the slides were washed stringently and the hybrids were detected by FITC-conjugated sheep-anti-digoxigenin (Roche, Manheim, Germany) and CYE3-conjugated avidine (Jackson Immuno research laboratories, Cambridgeshure, UK) Results were studied with a fluorescent microscope LSM700 Zeiss. Cells were pretreated in −20°C methanol/acetone for 20 min and the rest of the procedure for FISH on NCCIT cells was the same as above mentioned with omitting of the high pressure treatment.

### Xenografts generation

The NMRI/Nu-Nu strain of immune-compromised female mice (Mus musculus), aged 6–8 weeks (Charles River Laboratories, Wilmington, Massachusetts, USA) was used in this study. The animals were housed in the individually ventilated cages with sterile bedding, water, rodent chew feed and air. Experiments were carried out in accordance with the ARRIVE guidelines [45] and the animal research protocol was approved by the Institutional Animal Ethical Committee (DEC), Erasmus University Medical Center, Rotterdam, the Netherlands.

Six mice were used. The mice were anesthetized by the exposure to 2% isoflurane (Pharmachemie BV, Haarlem, The Netherlands) and placed ventral side up on a pre-warmed injecting pad. Approximately 10–12 million cells from each cell line suspended in 200 µl fresh culture medium with 20% serum were implanted under the skin with a 1-cc syringe and 24-gauge needle. Two week after implantation, xenograft tumor growth was checked by palpation and the size of xenografts was measured using a vernier calliper [xeno-tumor size (mm^2^)  =  length x width] twice a week and the mice were followed for a total period of maximal 12 months until they were sacrificed. For ethical reasons, primary xenograft tumors reaching the size of 225 mm^2^ were taken from study and sacrificed. The primary tumors were preserved by direct freezing in liquid nitrogen as well as by fixation in 10% formalin solution (Sigma-Aldrich, St. Louis, Missouri, USA) for subsequent histological analyses.

### Cultivation of NCCIT sub-lines, N-NCCIT and N2-NCCIT

N-NCCIT and N2-NCCIT were re-cultured similarly as described for the original NCCIT cell line.

## Results

### OCT3/4 and SOX2 in Embryonal Carcinoma (EC) cell lines

#### SOX2 amplification confirmed in NCCIT cells

**Figure 1:**
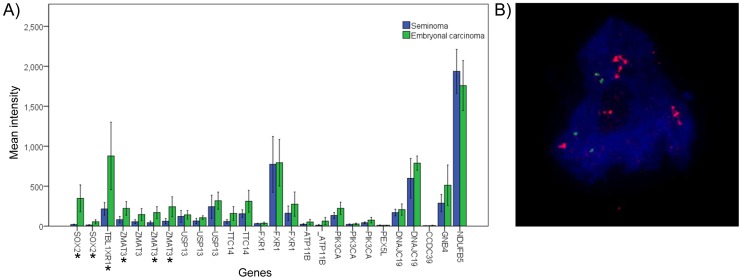
Gene expression within the amplified region. A) Expression histogram indicating the relative expression of genes mapped within the amplified region in a series of SE and EC. The stars indicate genes significantly differentially expressed between SE and EC (according to Mann Whitney U test). Most of the genes are represented by multiple specific probes; B) DNA-FISH result for SOX2 in NCCIT cells. A centromere 12 (C12) specific probe is used as control. Red dye (Cye3) shows SOX2 probe. For C12 probe green dye (FITC) is used. The blue background color is DAPi. Multiple red spots for SOX2 are detectable in each cell containing two green spots for C12, indicating SOX2 amplification. Magnification used was 630x. DNA-FISH was performed on cut tissue section with a thickness of 4 micron. This results in possible heterogeneity of the probe sizes detected. This issue was not a limitation as the purpose of this experiment was to determine the copy numbers and the size or intensity of the region. The FISH (BAC) probes were ordered at BACPAC Resources Center (BPRC) online:bacpac.chori.org. They were verified and confirmed at the department of genetics.

Our previous genome wide copy number investigation of multiple EC cell lines showed a specific amplification at the long arm of chromosome 3, band q23, including the SOX2 gene locus only in the NCCIT cell line [34]. The borders were defined as 177.604.206 bp (detected by RP11-71G7 probe) until 184.060.761 bp (detected by RP11-553E4 probe), encompassing a region of about 6.4 Mb. Various genes are mapped to this genomic fragment including SOX2 (Figure S1A & B). To investigate the pattern of expression of all genes within the amplified region in a series in primary GCC, including multiple pure EC, high throughput Affymetrix expression data analysis was performed, and compared to SE. The results showed a significant difference between the expression of SOX2 in EC and SE, being high versus low in expression respectively, which is in line with previous findings [19]. In total, 13 genes were analyzed in this region in which three genes, including SOX2, showed a significant difference between EC and SE (Figure 1A). Although there are other candidate genes within the amplified region, due to critical role of SOX2 in early development in close connection with OCT3/4 and its diagnostic value in the diagnosis of GCC, SOX2 was selected for further investigations. To verify the presence of SOX2 amplification in NCCIT, DNA FISH was performed using a verified probe for SOX2 gene (labeled with biotin). A centromere 12 (C12) specific probe (labeled with digoxigenin) was used in a double FISH experiment as control for copy number changes. The frequency of the signals obtained for SOX2 and C12 confirmed the amplification of SOX2, suggested to be at a single chromosome (Figure 1B).

#### Silencing OCT3/4 and SOX2 in NT2

To investigate the effect of reduced levels of OCT3/4 and SOX2 in the NT2 cell line, representative for pluripotent EC, without amplification of SOX2, siRNA-based OCT3/4 and SOX2 inhibition was performed. Based on the results obtained, specific siRNAs were chosen for further experiments (boxed in Figure S2). Because of the time effects, cells obtained 72 h after transfection were chosen for subsequent analysis. Both OCT3/4 and SOX2 RNA- knock-down in the NT2 cell line under these conditions have previously resulted in defined induction of differentiation as described before [33]. In the mentioned study, as a result of OCT3/4 downregulation, expression of pluripotency genes such as OCT3/4, SOX2, NANOG and LIN28 were down regulated and expression of some differentiation genes such as OTX1, PAX-6 (ectoderm), HAND1, BRACHYURY, MYOD1 (mesoderm), AFP, GATA4 (endoderm) and BMPR2 (germ cell) were upregulated.

Additionally, using the selected siRNAs, the microarray data on NT2 cells with OCT3/4 and SOX2 knockdown compared to the negative control NT2, showed 90% downregulation for OCT3/4 and 67% SOX2 knock-down on the RNA level 72 hours after the incubations. For microarray analysis Sentrix human6 BeadchipV3 was used. According to the array data, the number of intensity for OCT3/4 in the negative control NT2 was 8524 which decreased to 865 in the OCT3/4 knockdown NT2. SOX2 intensity in the negative control NT2 was 1460 which in SOX2 knock-down cells decreased to 479.

#### Silencing OCT3/4 and SOX2 in NCCIT

**Figure 2:**
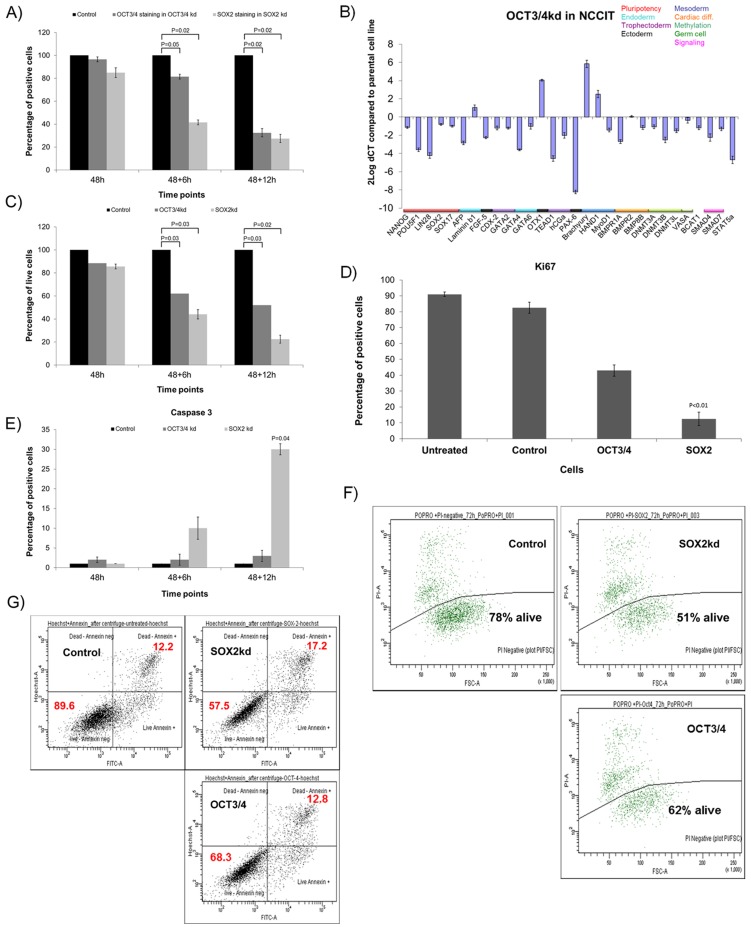
OCT3/4 and SOX2 knockdown in NCCIT. A) Percentage of positive cells for OCT3/4 and SOX2 in cells with reduced OCT3/4 and SOX2 levels compared to cells transfected with control siRNA in the NCCIT cells in three different time-points post transfection based on immunohistochemistry; The controls are set to 100 in all cases. B) Relative expression pattern of 32 genes representing targets for pluripotency and differentiation (ectoderm, mesoderm and endoderm). The expression levels are normalized based on the housekeeping gene *HPRT*; C) Percentage of living NCCIT cells with reduced level of OCT3/4 and SOX2 compared to cells transfected with control siRNA at each time point based on Trypan blue measurement; D) Percentage of positive NCCIT cells for Ki67 in untreated cells, cells transfected with control siRNA and cells with reduced levels of OCT3/4 and SOX2 at 48+12 h post transfection; E) Percentage of positive cells for Caspase 3 in untreated NCCIT cells, Cells transfected with control siRNA show a reduced level of OCT3/4 and SOX2 at all three time points after the transfection; F) FACS analysis with Propidium Iodide staining in cells transfected with control siRNA, cells transfected with SOX2 and OCT3/4 siRNA at 60 h post transfection. NCCIT cells transfected with control siRNA show the presence of 78% living cells, while cells transfected with SOX2 siRNA show 51% living cells, NCCIT cells with OCT3/4kd show 62% live cells. G) Annexin V assay results in cells with siRNA control and cells with reduced SOX2 and OCT3/4 at 60 h after transfection. In the control, percentages of living-annexin negative cells are 89.6%, while dead-annexin positive cells are 12.2%. In SOX2kd cells, the amount of living-annexin negative cells is 57.5% while the amount of dead-annexin positive cells is 17.2%. In cells transfected with OCT3/4 siRNA, living-annexin negative cells are 68.3% while dead-annexin positive cells are 12.8%. The differences between the percentages of live and dead cells in all experiments are due to using independent and various methods in order to prove apoptosis and different sensitivity of the materials and methods used. As it is demonstrated, OCT3/4 knock-down also induces apoptosis, however, this effect is not as dramatic as SOX2 knock-down. The FACS analysis were performed in triplicate.

To investigate the effect of reduced levels of OCT3/4 and SOX2 in the NCCIT cell line, as done in the NT2 cell line, the selected siRNAs for OCT3/4 and SOX2 (see above) were transfected, and the cells were investigated at three different time points (48, 48+6 h and 48+12 h). Use of each siRNA specifically led to a down-regulation of OCT3/4 and SOX2 protein expression in NCCIT. The percentage of positive cells for these proteins was measured in negative control cells and cells transfected with siRNAs, by immunohistochemistry, shown to be an informative method for analysis [33] Additional data are shown in Figure S3. The results indicate that the expression level of OCT3/4 and SOX2 protein expression were reduced significantly in time (Figure 2A).

#### OCT3/4-down-regulation in NCCIT results in loss of pluripotency

To investigate the effect of OCT3/4 down-regulation on the identity of the NCCIT cells, expression level of a selected panel of genes representative for pluripotency and differentiation (for all embryonic germ layers: mesoderm, endoderm and ectoderm) was measured using q-RT-PCR, as reported before [33]. As in the NT2 cells, OCT3/4 down-regulation resulted in differentiation, demonstrated by loss of the pluripotency factors (*OCT3/4*, *NANOG* and *LIN28*) and up-regulation of some differentiation genes, including *OTX1* (ectoderm), *brachyury* and *HAND1* (mesoderm), and *LAMB1* (endoderm) (Figure 2B).

The details on qRT-PCR panel has been described in [46]. No morphological changes were been observed in the cells under investigation.

#### SOX2-siRNA caused major apoptosis in NCCIT

As reported for OCT3/4, reduction of SOX2 expression in NT2 resulted in induction of differentiation [33]. In contrast, in the NCCIT cell line SOX2 reduction did not result in differentiation, but instead, it caused a progressive loss of cells in time (48, 48+6, 48+12 and 72 hours after transfection). Cell death was obvious after 54 hours and prominent at 60 hours. Because at the last time point, almost no viable cells were present, no further analysis could be done including expression profiling. Using Trypan blue staining, the presence of live cells was measured at each time point (Figure 2C). The results demonstrate at the latest time point after transfection a progressive and significant effect of SOX2 and OCT3/4 reduction on the amount of living cells in time, indicating only 20% living cells in the SOX2 reduced cells and 50% living cells in the OCT3/4 reduced cells. To investigate the effect of SOX2 and OCT3/4 down-regulation on proliferation, immunohistochemical staining was performed for Ki67 on cytospin slides at the latest time point (60 h). The results (Figure 2D) demonstrate that OCT3/4, inducing loss of pluripotency (see above), also resulted in a decrease in proliferation status compared to the negative siRNA control and untreated cells for about 50%. The effect of SOX2 reduction was even more severe, resulting in about 10% positive cells (p<0.01). Subsequently, we investigated whether loss of cells was also the result of increased apoptosis, for which various approaches were used. These include immunohistochemistry for the apoptosis marker Caspase 3 (Figure 2E). Positive staining was found predominantly and significantly in the cells with SOX2 inhibition, especially at the 60 hours time point (p = 0.04). In contrast, inhibition for OCT3/4 resulted in less than 4% apoptotic cells. This pattern was confirmed by FACS analysis using Propidium Iodide (PI) exclusion test and Annexin V staining (Figure 2F, G). These data demonstrate that the major decrease in the amount of living NCCIT cells due to reduced SOX2 expression is explained by induction of apoptosis, as determined by independent methods.

#### N-NCCIT and N2-NCCIT xenografts show a consistent positive expression for SOX2

**Figure 3:**
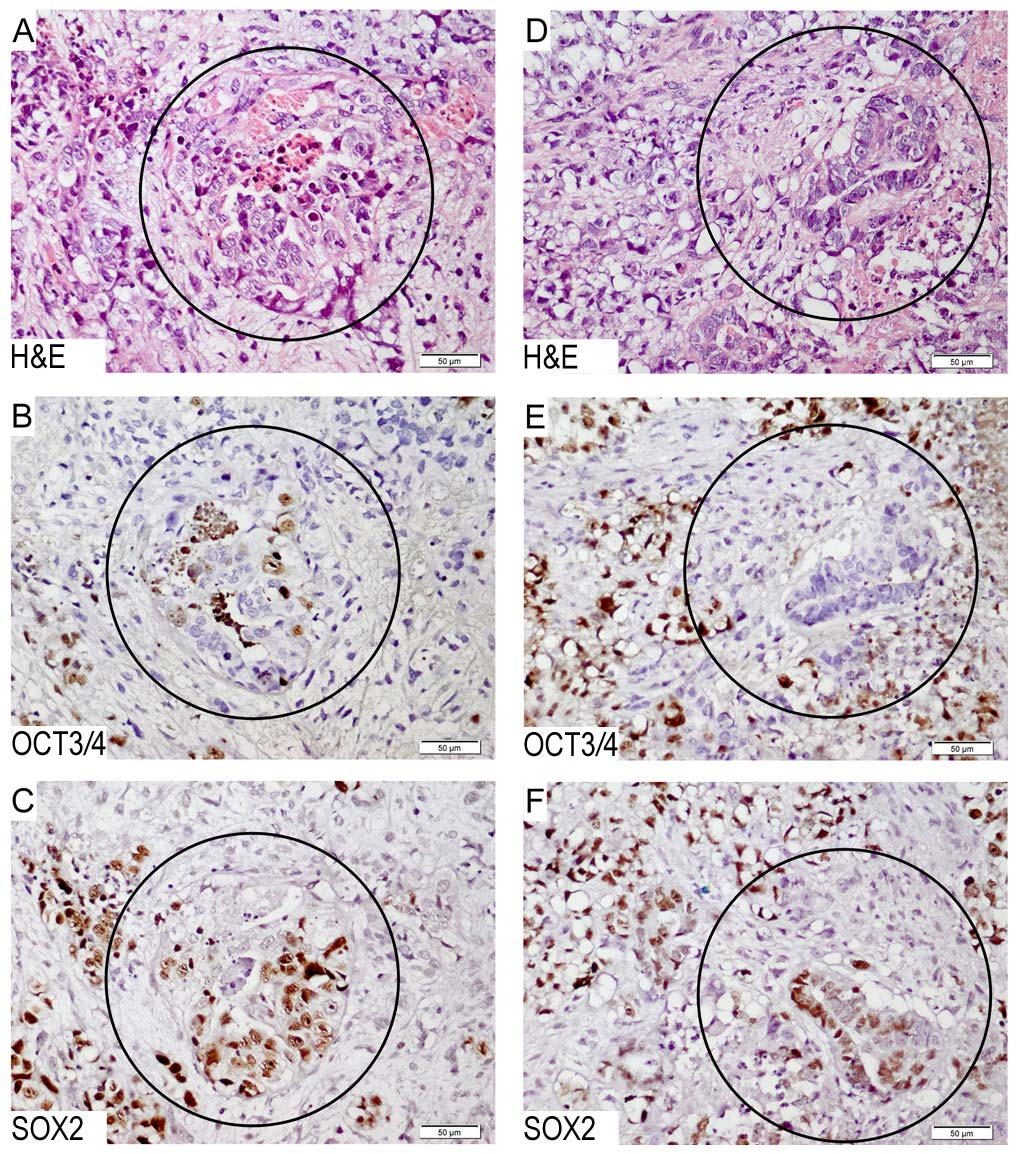
Immunohistochemistry for SOX2 and OCT3/4 on nude mice xenografts of NCCIT. Images A, B and C belong to N-NCCIT; D, E and F belong to N2-NCCIT. A & D) H & E staining demonstrates the histological composition, showing regions with differentiation (indicated with a circle); B & E) Staining for OCT3/4 showing the presence of heterogeneity, showing undifferentiated (positive) and differentiated (negative) cells; C & F) Staining for SOX2 shows that the malignant cells are consistently positive, in spite of the presence or absence of OCT3/4.

It is known that NCCIT has the capacity to differentiate [31], [47], in line with the results obtained from the OCT3/4 inhibition experiments (see above). SOX2 is found to be an absolute marker for EC in combination with OCT3/4, although it can also be found more heterogeneously in differentiated components, especially teratoma [19]. In the context of the induction of apoptosis by means of SOX2 suppression in NCCIT, it is interesting to investigate whether NCCIT cells grown as a xenograft *in vivo* remains SOX2 positive throughout, in spite of possible differentiation. To answer this question, multiple mice xenografts were generated from the parental NCCIT cell line (referred to N- and N2-NCCIT). The established tumors were characterized, and a sub-line was subsequently cultured continuously *in vitro.* The N-NCCIT sub-line was tested for OCT3/4 and showed positive staining (Figure S4). The *in vivo* tumors were tested for SOX2 and OCT3/4. The overall pattern (representative examples shown in Figure 3) indicated that in spite of morphological induction of differentiation, supported by loss of OCT3/4 by immunohistochemistry, this was not accompanied by loss of the expression of SOX2. In other words, SOX2 remained positive in all components of the tumors.

### Detection of amplification for SOX2 and OCT3/4 in EC

Because *SOX2* is amplified in the EC cell line NCCIT, with a functional effect on survival, it is of interest to check whether primary GCC, have *SOX2* amplification. Particularly, the so-called nullipotent EC might be of interest to be investigated, although this component might also be present in mixed nonseminomas, i.e., tumors with a histologically mixed composition. A series of 12 pure EC and 34 mixed GCCs including an EC component were studied using the double FISH method (see above). No amplification for *SOX2* or *OCT3/4* (i.e. more than 6 copies) was found in the cases investigated (shown in Figure S5).

## Discussion

Pluripotent stem cells have been isolated from a variety of human and mouse sources as models to investigate processes involved in early embryonal development [48], [49]. Two of the well-studied cell types are ES cells derived from the inner cell mass of blastocyst-stage embryos and EC cells, the nonseminomatous stem cells of GCC [49], [50]. By definition, pluripotent stem cells have extensive self-renewal capacity and the ability to differentiate into wide variety of cell types [48], [49], [51]. In fact EC cells derived from the progenitor of the germ line are a malignant equivalent of ES cells [52], thus they provide a good model to study early embryonal development as well as tumorigenesis [49].

OCT3/4 and SOX2 are transcription factors essential to the pluripotent and self-renewing phenotypes of ES cells. These master ES cell pluripotency factors are highly expressed in EC [19], [53]. Representative cell lines of EC which are capable of differentiation (NT2, NCCIT, 2102Ep) have been generated and extensively used for studies [30], [31], [47], [50]. Based on our molecular data derived from array CGH data, the NCCIT cell line, derived from an extra-gonadal GCC, shows a restricted amplification of the 3q23 region. This region contains *SOX2*, amongst many other genes. Here we demonstrate that indeed *SOX2* maps within the minimal region of overlap of the amplification. This triggered a more detailed analysis of the role of SOX2 in this cell line compared to an EC cell line without such a genomic amplification. In fact, *SOX2* amplification and its pathogenic role association with oncogenesis have been reported in human lung squamous cell carcinoma [54]. In addition, targeting SOX2 in breast cancer cell lines have shown that siRNA-mediated knock-down of SOX2 resulted in cell cycle arrest by down-regulation of Cyclin D1 and this arrest in cell cycle was accompanied by an inhibition of tumor cell proliferation in xenograft models [18]. Most recently, it has been reported that inhibition of SOX2 might be of therapeutic potential for EC [29]. In that particular study, SOX2 down-regulation in the EC cell line NEC8, when established *in vivo*, induced tumor growth suppression in case of a limited tumor size. In contrast, down-regulation showed no effect on progression in case of a tumor of larger size. This is likely related to loss of SOX2 expression to some extent and differentiation. This study indicates that suppression of the SOX2 expression might be useful to block cell proliferation in early stage EC. Interestingly, as a result of our array CGH study on multiple EC cell lines, NCCIT cell line showed amplification of the long arm of chromosome 3, band q23 including the SOX2 gene locus [34].

Unlike the majority of GCC cell lines which are derived from testicular cancers, NCCIT is derived from an extragonadal mediastinal GCC. In 1988, NCCIT was established as an *in vitro* cell line, composed of developmentally pluripotent cells capable of somatic and extra-embryonic differentiation. Nude mouse-xenografts of NCCIT contained foci of EC, yolk sac tumor, immature somatic tissues, and trophoblastic giant cells indicating that this cell line is indeed developmentally pluripotent [47]. In 1993, retinoic-induced differentiation of NCCIT into all three embryonic germ layers and extra-embryonic cell lineages was reported, and in fact, it was suggested that the parental NCCIT cells show characteristics intermediate between SE and EC [31]. We investigated the effect of OCT3/4 and SOX2 down-regulation in NCCIT. The results demonstrate that inhibition of OCT3/4 resulted in loss of pluripotency and only partial apoptosis, while inhibition of SOX2 led to extensive cell death. This suggests that survival of NCCIT is dependent on the presence of SOX2 expression, referred to as oncogene-dependence [55]. This was supported by various methods and read out systems. As a control, inhibition of OCT3/4 and SOX2 was done similarly in the NT2 cell line, which showed induction of differentiation under the experimental conditions applied [33]. The next step in our study was to investigate the potential of the NCCIT cells to undergo differentiation *in vivo*. Therefore multiple xenografts were generated. Interestingly, in spite of induction of partial differentiation, supported by loss of OCT3/4, all tumor cells remained positive for SOX2. This is in line with the hypothesis that differentiation is possible, as found in the *in vitro* experiments, even in spite of SOX2 amplification. The lineages formed are selected by continuous expression of SOX2 [56]. In this context the potential use of inhibition of SOX2 in a clinical setting, should be considered carefully because down-regulation of SOX2 might result in the induction of differentiation, leading to potentially highly metastatic clones.

The absence of SOX2 amplification in a series of *in vivo* pure EC and EC containing mixed nonseminomas, suggest that SOX2 oncogene dependence, at least due to gene amplification, is not a frequent mechanism in GCC, which questions indeed the approach of targeted therapy in a clinical setting. In this context it might be of interest to check whether the NEC8 cell line contains SOX2 amplification [29]. In addition,as the number of EC tumors investigated for SOX2 amplification (50 samples) is not a large number, there is a possibility that SOX2 amplification is present in a limited number of cases.

**Figure 4:**
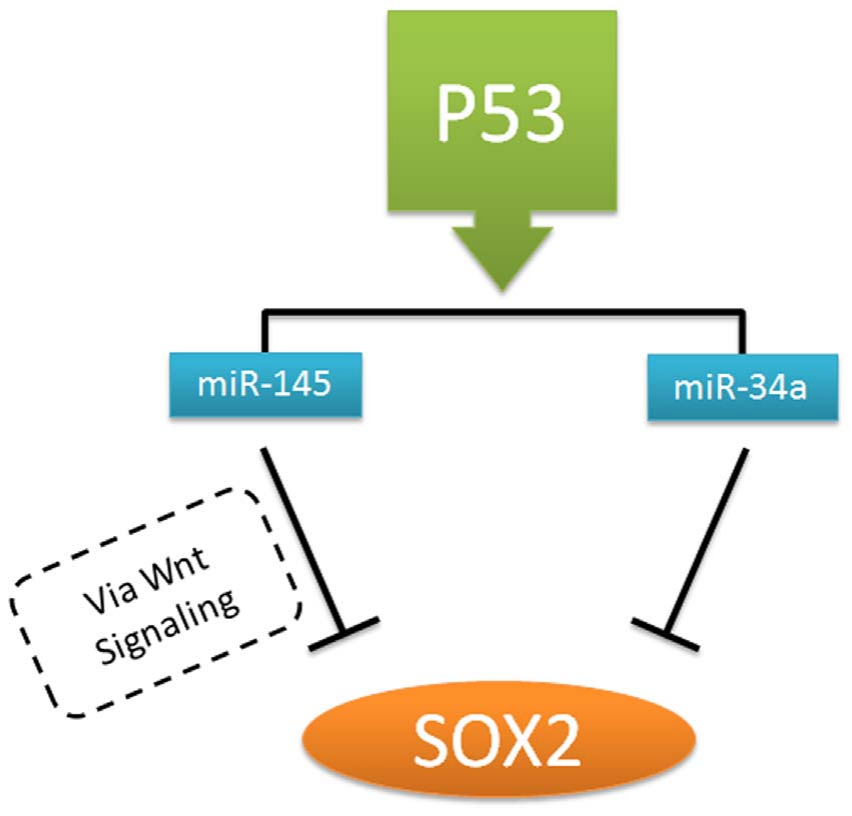
Schematic representation of the hypothetical indirect regulation of SOX2 by p53 status. Wild type p53 results in induction of expression of miR-145 and miR-34a. Subsequently, miR-34a and mir-145 down-regulates pluripotency genes, including SOX2, the latter via Wnt signaling.

The presence of SOX2 amplification in the NCCIT cell line might be explained by involvement of other mechanisms, like absence of a functional p53 status. The importance of the connection between pluripotency genes, ES pluripotency stem cell miRs and wildtype/mutant p53 in the pathogenesis of GCC has been previously described [57]. It has been shown that p53 activates expression of miR-34a and miR-145, which in turn repress key stem cell factors such as OCT3/4 and SOX2 to prevent self-renewal and promote differentiation [58] (Figure 4). miR-145 regulates such activities by activation of WNT signaling pathway via intracellular localization of β-catenin [59]. Indeed, earlier miR profiling experiments demonstrate that miR-34a is not present in NCCIT, and miR-145 shows a low level of expression [60]. This might be due to the mutated p53 status, resulting in loss of inhibition of SOX2 expression. Additionaly, in this context it was shown that miR-371-3 which is thought to mimic the role of mutant p53 in GCC is expressed at low levels in NCCIT with mutant p53 and in NT2 with low expression level of wild type p53 [35].

Interestingly, NT2 and TCam2 (another GCC cell line with seminomatous characteristics) show similar corelation patterns between p53 status and expression levels of miR-34a and miR-145. NT2 has a low level of wild type p53 as well as miR-34a and miR-145, with a high level of SOX2. In contrast, the TCam2 cells have a relatively normal level of wild type p53, and a high level of miR-34a and miR-145. Indeed, as expected no SOX2 is expressed, in line with the seminoma type of cells, linked to expression of SOX17 instead of SOX2. This justifies the absence of WNT signaling in TCam2, since SOX17 is known to suppress this particular pathway [61], [62]. This connection between p53, its downsteam targets regulating the expression of pluripotency factors such as SOX2 is of interest since most GCC patients have low levels of wild type p53, and p53 mutations are rarely observed [63]. We have shown that wild type p53 status is related to the response of GCC to DNA damaging agents, including cisplatin [63]. This can elegantly explain why GCC show such an exceptional sensitivity to DNA damaging agents [64].

Most recently it has been shown that site-specific phosphorylation of OCT3/4 regulated by AKT, promotes stemness of EC cells (i.e., NCCIT) compared to ES cells [65]. Presence of this site-specific phosphorylation promotes release of the OCT3/4 protein from the *AKT1* promoter, resulting in induced expression, which will lead to suppression of apoptosis, and simultaneously, enhances capacity of OCT3/4 to form a complex with NANOG and SOX2, promoting pluripotency. This additional effect promotes tumorigenic capacity of EC compared to ES, which might be of interest for further investigation.

In conclusion, these data shed novel light on the role of ES cell pluripotency factors in NCCIT (as an EC cell line) which can reflect the role of these factors, particularly SOX2 and its dependence in relation to differentiation, in the etiology of EC and regulation of stem cell differentiation.

## Supporting Information

Figure S1 **Genomic region of amplification.** A) UCSC genome browser (version hg19) representation of the genomic region of amplification at the long arm of chromosome 3, band q23.33, in NCCIT cells. The borders are 177.604.260 bp and 184.060.761 bp (encompassing a region of about 6.4 Mb). The genes mapped to this region are shown including SOX2 locus; B) Array CGH result, the region of amplification in chromosome 3q is indicated in red circle, the borders are defined between the probes RP11-71G7 and RP11-553E4, respectively. The y axis indicates the unique position number based on probe distribution and the X axis shows a log ratio compare to normal sample.
(TIF)

Figure S2 **Western blot analysis of down-regulation of OCT3/4 and SOX2 in NT2 cells at various time points (24, 48, 72, 96 and 120 hours).** A) NT2 cells are transfected with two independent OCT3/4 siRNAs (“Matin” and “Hay”), two independent β-actin siRNAs and negative control siRNA. OCT3/4 “Hay” is selected for further experiments. B) NT2 cells are transfected with three independent SOX2 siRNAs (13294, 13295 and 13296), one β-actin siRNA and negative control siRNA. SOX2-13294 siRNA is selected for further experiments. The selected siRNAs are boxed in red within the Figure. These conditions showed the most profound down-regulation of expression at the protein level (over 90%) (In 72 h incubation, SOX2- siRNA 13295 and 13296 have been switched).
(TIF)

Figure S3 **Silencing OCT3/4 and SOX2 in NCCIT. Examples of immunohistochemistry on cytospin slides.** A) SOX2 staining in negative control NCCIT. B) OCT3/4 staining in negative control NCCIT. C) SOX2 staining in SOX2kd NCCIT cells. D) OCT3/4 staining in OCT3/4kd NCCIT cells.
(TIF)

Figure S4 **OCT/4 staining for cultivated N-NCCIT cells.** Brown colored cells show 95% positive staining for OCT3/4 in cultivated N-NCCIT cells (sub-line of NCCIT cells). Magnification used was 100x.
(TIF)

Figure S5 **Examples of FISH for SOX2 on EC tumors.** Red dye (Cye3) shows SOX2 probe. For C12 probe green dye (FITC) is used. Not more than two copies of SOX2 probe in each nuclease are detected in these tumors.
(TIF)
