# Supplementary figures and images for: Role of SOX2 in the Etiology of Embryonal Carcinoma, Based on Analysis of the NCCIT and NT2 Cell Lines
Source: PLoS One. 2014 Jan 3;9(1):e83585. doi: 10.1371/journal.pone.0083585 (PMC3880257; doi:10.1371/journal.pone.0083585)

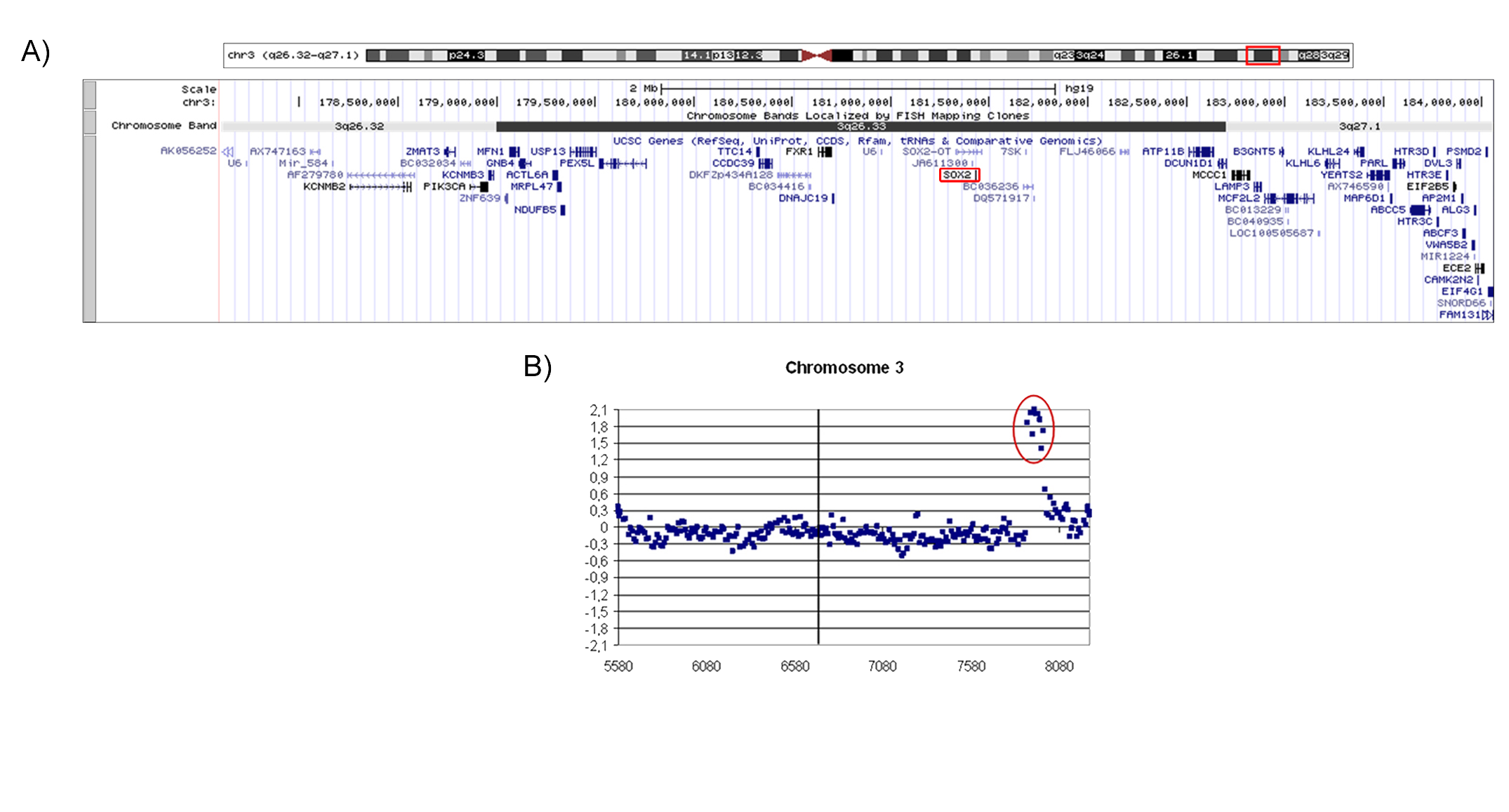

Supplement: Figure S1 — Genomic region of amplification. A) UCSC genome browser (version hg19) representation of the genomic region of amplification at the long arm of chromosome 3, band q23.33, in NCCIT cells. The borders are 177.604.260 bp and 184.060.761 bp (encompassing a region of about 6.4 Mb). The genes mapped to this region are shown including SOX2 locus; B) Array CGH result, the region of amplification in chromosome 3q is indicated in red circle, the borders are defined between the probes RP11-71G7 and RP11-553E4, respectively. The y axis indicates the unique position number based on probe distribution and the X axis shows a log ratio compare to normal sample. (TIF) [file pone.0083585.s001.tif]

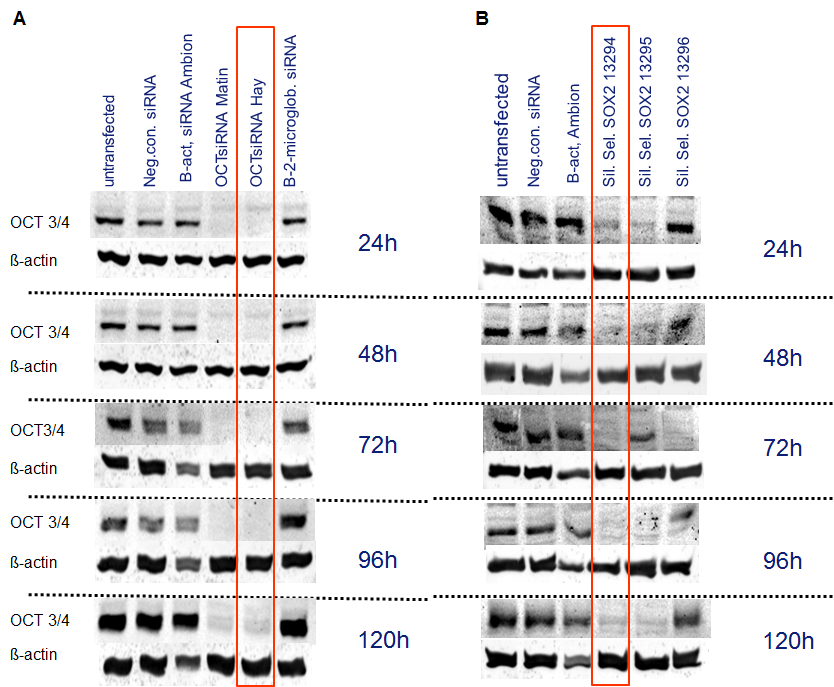

Supplement: Figure S2 — Western blot analysis of down-regulation of OCT3/4 and SOX2 in NT2 cells at various time points (24, 48, 72, 96 and 120 hours). A) NT2 cells are transfected with two independent OCT3/4 siRNAs (“Matin” and “Hay”), two independent β-actin siRNAs and negative control siRNA. OCT3/4 “Hay” is selected for further experiments. B) NT2 cells are transfected with three independent SOX2 siRNAs (13294, 13295 and 13296), one β-actin siRNA and negative control siRNA. SOX2-13294 siRNA is selected for further experiments. The selected siRNAs are boxed in red within the Figure. These conditions showed the most profound down-regulation of expression at the protein level (over 90%) (In 72 h incubation, SOX2- siRNA 13295 and 13296 have been switched). (TIF) [file pone.0083585.s002.tif]

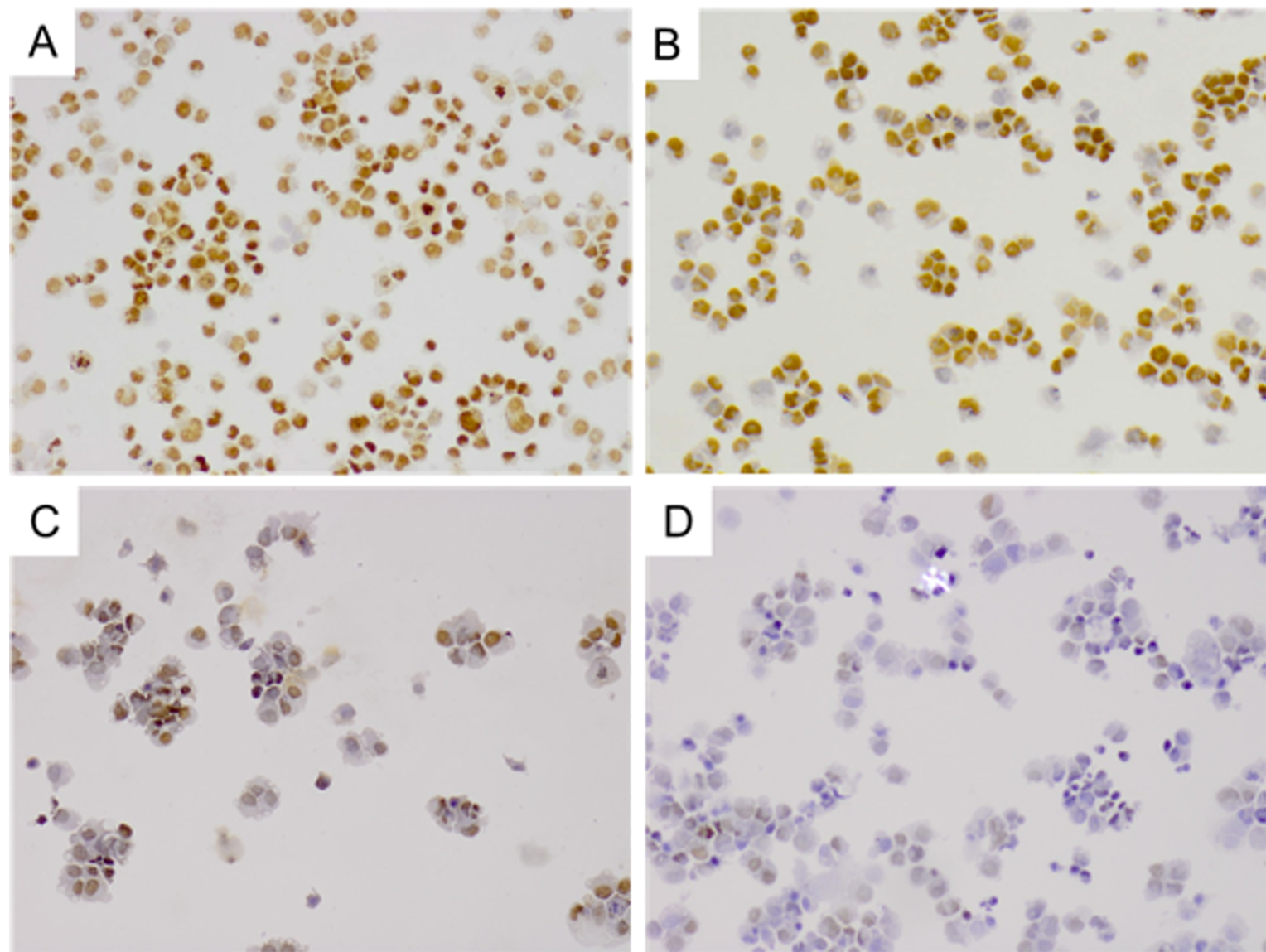

Supplement: Figure S3 — Silencing OCT3/4 and SOX2 in NCCIT. Examples of immunohistochemistry on cytospin slides. A) SOX2 staining in negative control NCCIT. B) OCT3/4 staining in negative control NCCIT. C) SOX2 staining in SOX2kd NCCIT cells. D) OCT3/4 staining in OCT3/4kd NCCIT cells. (TIF) [file pone.0083585.s003.tif]

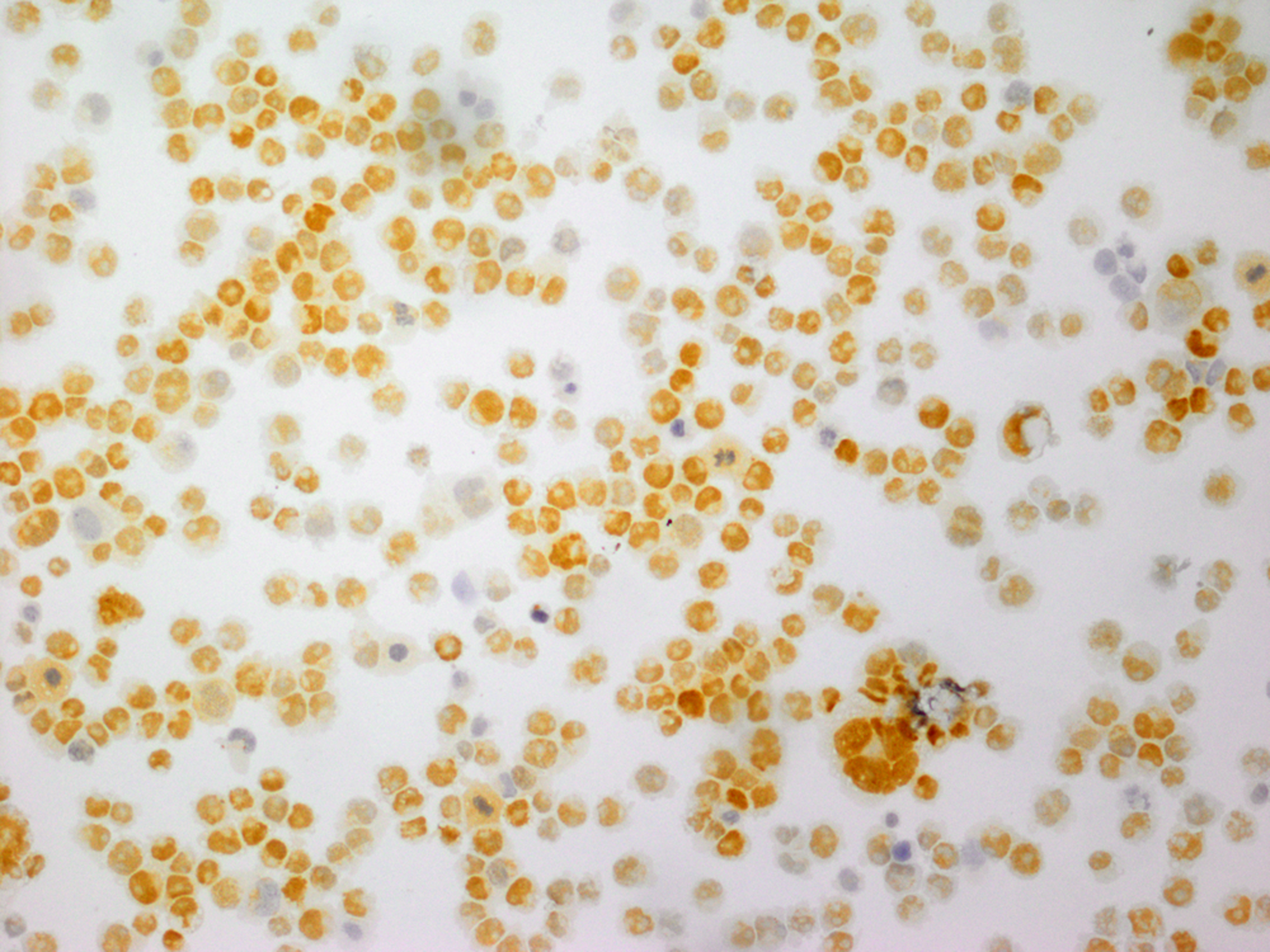

Supplement: Figure S4 — OCT/4 staining for cultivated N-NCCIT cells. Brown colored cells show 95% positive staining for OCT3/4 in cultivated N-NCCIT cells (sub-line of NCCIT cells). Magnification used was 100x. (TIF) [file pone.0083585.s004.tif]

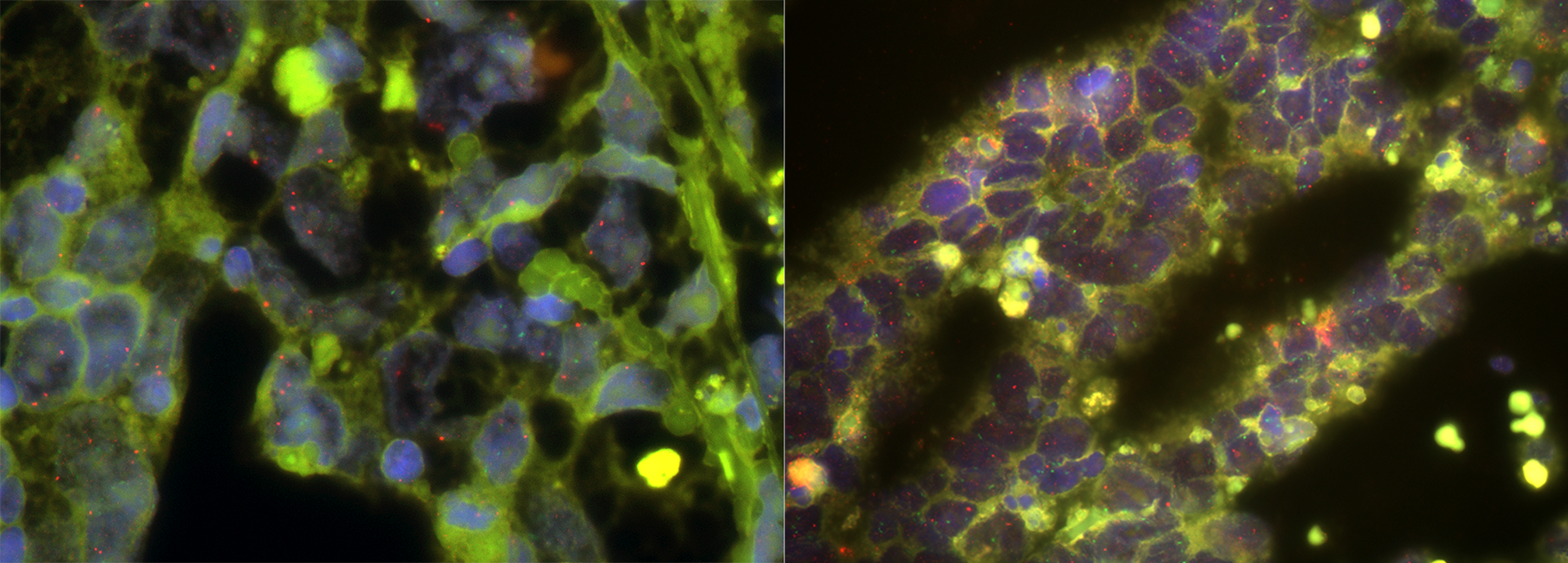

Supplement: Figure S5 — Examples of FISH for SOX2 on EC tumors. Red dye (Cye3) shows SOX2 probe. For C12 probe green dye (FITC) is used. Not more than two copies of SOX2 probe in each nuclease are detected in these tumors. (TIF) [file pone.0083585.s005.tif]
